# The Impact of Prematurity at Birth on Short-Term Postoperative Outcomes Following Posterior Spinal Fusion for Adolescent Idiopathic Scoliosis

**DOI:** 10.3390/jcm12031210

**Published:** 2023-02-03

**Authors:** Neil V. Shah, Marine Coste, Adam J. Wolfert, Samuel Gedailovich, Brian Ford, David J. Kim, Nathan S. Kim, Chibuokem P. Ikwuazom, Neil Patel, Amanda M. Dave, Peter G. Passias, Frank J. Schwab, Virginie Lafage, Carl B. Paulino, Bassel G. Diebo

**Affiliations:** 1 Department of Orthopaedic Surgery and Rehabilitation Medicine, State University of New York (SUNY) Downstate Health Sciences University, Brooklyn, NY 11203, USA; 2 Department of General Surgery, Icahn School of Medicine at Mount Sinai, New York, NY 10029, USA; 3 Department of Orthopaedic Surgery, University of Connecticut Health Center, Farmington, CT 06030, USA; 4 Department of Pediatrics, University of Nebraska Medical Center, Omaha, NE 68198, USA; 5 Department of Orthopedic Surgery, NYU Langone Orthopedic Hospital, New York, NY 10010, USA; 6 Department of Orthopaedic Surgery, Lenox Hill Hospital, Northwell Health, New York, NY 10075, USA; 7 Department of Orthopaedic Surgery, NewYork-Presbyterian Brooklyn Methodist Hospital, Brooklyn, NY 11215, USA; 8 Department of Orthopaedic Surgery, Warren Alpert School of Medicine, Brown University, East Providence, RI 02903, USA

**Keywords:** adolescent idiopathic scoliosis, prematurity, posterior spinal fusion, complications, short-term outcomes

## Abstract

Prematurity is associated with surgical complications. This study sought to determine the risk of prematurity on 30-day complications, reoperations, and readmissions following ≥7-level PSF for AIS which has not been established. Utilizing the American College of Surgeons National Surgical Quality Improvement Program (ACS NSQIP)-Pediatric dataset, all AIS patients undergoing ≥7-level PSF from 2012–2016 were identified. Cases were 1:1 propensity score-matched to controls by age, sex, and number of spinal levels fused. Prematurity sub-classifications were also evaluated: extremely (<28 weeks), very (28–31 weeks), and moderate-to-late (32–36 weeks) premature. Univariate analysis with post hoc Bonferroni compared demographics, hospital parameters, and 30-day outcomes. Multivariate logistic regression identified independent predictors of adverse 30-day outcomes. 5531 patients (term = 5099; moderate-to-late premature = 250; very premature = 101; extremely premature = 81) were included. Premature patients had higher baseline rates of multiple individual comorbidities, longer mean length of stay, and higher 30-day readmissions and infections than the term cohort. Thirty-day readmissions increased with increasing prematurity. Very premature birth predicted UTIs, superficial SSI/wound dehiscence, and any infection, and moderate-to-late premature birth predicted renal insufficiency, deep space infections, and any infection. Prematurity of AIS patients differentially impacted rates of 30-day adverse outcomes following ≥7-level PSF. These results can guide preoperative optimization and postoperative expectations.

## 1. Introduction

Despite a slight decline in prevalence in recent decades, more than 10% of births in the United States continue to be preterm, defined as a birth that occurs before 37 weeks of gestation [1]. Prematurity has a well-delineated association with increased rates of comorbidities and complications in childhood and throughout life, including, but not limited to, higher rates of cardiovascular, respiratory, and behavioral/cognitive conditions as well as increased morbidity and mortality in the neonatal period [1,2,3,4,5,6,7,8]. The management of premature patients must take into consideration challenges that are not as relevant to individuals born at term. Understanding and accounting for such differences can allow providers to better optimize the health of patients with a history of premature birth when planning for surgical procedures later in life.

Adolescent idiopathic scoliosis (AIS) is the most common form of scoliosis and is defined as idiopathic scoliosis in individuals 10–18 years old [9]. Little is known about the exact etiology of AIS, although studies suggest a multi-factorial origin [10,11,12,13,14]. Existing literature has reported a prevalence between 2 and 9.2%, although only approximately 0.23% of patients end up requiring surgical treatment [15,16]. Operative treatment is performed with spinal fusion through either an anterior, posterior, or combined approach, with posterior spinal fusion (PSF) with instrumentation identified as the gold standard [17,18]. AIS has been shown to have a higher incidence among individuals born prematurely. Premature populations also generally experience higher complication rates when undergoing corrective surgeries for congenital defects [11,19,20,21,22]. Despite this, the impact of prematurity on surgical outcomes, specifically in AIS patients, is not well-documented.

The purpose of this study was to explore the association between history of prematurity at birth and short-term surgical outcomes in AIS individuals undergoing PSF. Additionally, the impact of degree of prematurity at birth, along with differences across prematurity groups, were assessed with respect to short-term surgical outcomes, with the hypothesis that prematurity, as well as increasing degree of prematurity of birth, would be respectively associated with an increased incidence of short-term postoperative adverse outcomes.

## 2. Materials and Methods

The American College of Surgeons National Surgical Quality Improvement Program (ACS NSQIP)-Pediatric dataset was queried to identify AIS patients who underwent ≥7-level PSF from 2012–2016, using the International Classification of Diseases (ICD-9) code 737.30 and the Current Procedural Terminology (CPT) codes 22,802 and 22,804. The NSQIP variable “PREM_BIRTH” allowed for the identification of patients born before 37 weeks of gestation. Patients from this subset of all operative AIS patients were subsequently propensity score-matched to a cohort of full-term (≥37 weeks) individuals for age, sex, and number of spinal levels fused. Additionally, premature patients were further stratified by World Health Organization (WHO) preterm categories: extremely premature (<28 weeks), very premature (28 ≤ x < 32 weeks), moderate-to-late premature (32 ≤ x < 37 weeks), and term (≥37 weeks).

Age, sex, race, and baseline comorbidities were identified and compared between cohorts. Comorbidities evaluated included history of asthma, bronchopulmonary dysplasia/chronic lung diseases, structural pulmonary/airway abnormalities, esophageal/gastric/intestinal diseases, previous cardiac surgery, developmental delay/impaired cognitive status, seizure disorders, cerebral palsy, structural central nervous system abnormalities, and neuromuscular disorders. Cohorts were also compared for hospital-related and intraoperative variables, including total operative time and length of stay. Rates of complications, readmissions, and reoperations within the 30-day postoperative period were compared. Complications evaluated included superficial surgical site infections (SSI), wound dehiscence, deep space infections, pneumonia, urinary tract infection (UTI), renal insufficiency, transfusion, stroke/cerebrovascular accident, cardiac arrest, pulmonary embolism (PE), unplanned reintubation, acute renal failure, and sepsis.

Comparisons of baseline characteristics, hospital-related parameters, and readmission, reoperation, and complication rates between the premature and term cohorts were computed using appropriate parametric tests. Multivariate logistic regression evaluated independent outcome predictors utilizing prematurity group, age, sex, race, and individual baseline comorbidities as covariates. Univariate analysis with post hoc Bonferroni compared demographics, hospital parameters, and 30-day outcomes. All statistical calculations were performed in SPSS version 24.0 (IBM Corp., Armonk, NY, USA), and the threshold for statistical significance was set at *p* < 0.05.

## 3. Results

### 3.1. Demographics

A total of 5531 patients were included for analysis, of which 5099 (92.2%) were born at term and 432 (7.8%) were born premature. Of the 432 patients born premature, 250 (57.9%) were moderate-to-late premature, 101 (23.4%) were very premature, and 81 (18.8%) were extremely premature. Following propensity score-match, cohorts of 432 premature patients and 432 term patients were analyzed. Premature and term cohorts did not differ significantly by age, sex, and race. The average age was 14.1 years in the premature cohort and 14.4 years in the term cohort (*p* = 0.071) (Table 1). The premature cohort was 67.6% females compared to 64.4% females in the term cohort (*p* = 0.315). The premature cohort was 76.5% White and 21.8% Black, while the term cohort was 80.1% Black and 16.9% White (*p* = 0.109) (Table 1).

### 3.2. Comorbidities and Hospital-Related Parameters

Significant differences in the rates of baseline comorbidities between the two cohorts were observed. Premature patients had significantly greater rates of neuromuscular disease (30.1% vs. 22.7%), asthma (12.5% vs. 5.8%), bronchopulmonary dysplasia/chronic lung disease (7.4% vs. 1.9%), esophageal/gastric/intestinal disease (15.0% vs. 7.2%), previous cardiac surgery (7.6% vs. 1.4%), seizure disorder (14.8% vs. 5.3%), structural CNS abnormality (19.7% vs. 7.6%), cerebral palsy (22.7% vs. 4.9%), and cognitive impairment (36.3% vs. 11.6%), all *p* < 0.05 (Table 2).

Evaluating for hospital-related parameters, the premature cohort had a significantly higher average American Society of Anesthesiologists (ASA) classification than the term cohort (ASA III: 36.1% vs. 19.0%, respectively; *p* < 0.001), as well as a longer average length of stay (6.5 days vs. 5.6 days, respectively; *p* = 0.045) (Table 3). There was no difference in operative time (premature: 305.1 vs. term: 296.3 min, *p* = 0.252) or fusion length (premature: 43.3% vs. term: 39.4% ≥13-level fusions, *p* = 0.240) between the two cohorts.

When comparing the 432 premature patients across their various degree of prematurity subgroups to the 5099 patients born at term, extremely premature patients had significantly higher baseline rates of the following comorbidities than very premature, moderate-to-late premature, and term patients, respectively (Table 4): neuromuscular disorder (44.4% vs. 35.6%, 23.2%, and 14.5%), esophageal/GI disease (23.5% vs. 14.9%, 12.4%, and 5.4%), structural CNS abnormalities (32.1% vs. 24.8%, 13.6%, and 8.9%), cerebral palsy (45.7% vs. 31.7%, 11.6%, and 4.0%), cognitive impairment (58.0% vs. 41.6%, 27.2%, and 11.0%), asthma (19.8% vs. 12.9%, 10.0%, and 7.3%), chronic lung disease (19.8% vs. 4.0%, 4.8%, and 1.6%), and structural pulmonary/airway abnormalities (9.9% vs. 4.0%, 6.8%, and 3.0%; all *p* < 0.001). In the extremely premature cohort, 51.9% of patients were ASA Class 3, while the majority of patients in each of the very premature, moderate-to-late premature, and term cohorts were ASA I or II (*p* < 0.001). The proportion of patients who underwent ≥13-level fusions progressively increased with the degree of prematurity, as the term cohort underwent ≥13-level fusions for 30.2% of PSFs, while the moderate-to-late premature, very premature, and extremely premature patients underwent ≥13-level fusions 40.0%, 43.6%, and 53.1% of the time, respectively (*p* < 0.001). By extension, the operative time also increased with the degree of prematurity, with the average PSF taking 278.2 min in the term cohort, and taking 295.2, 310.2, and 329.3 min in the moderate-to-late premature, very premature, and extremely premature cohorts, respectively (*p* < 0.001).

### 3.3. Outcomes

When comparing the propensity score-matched cohorts of 432 premature and 432 term patients, premature patients experienced significantly higher rates of readmission within the 30-day postoperative period than patients born at term (11.0% vs. 3.2%; *p* = 0.003) (Table 3).

There was, however, not a significant difference in 30-day postoperative complication rate (premature: 6.3% vs. term: 4.2%, *p* = 0.168) or reoperation rate (premature: 4.6% vs. term: 2.3%, *p* = 0.063) between these two cohorts. Amongst complications, premature patients had higher rates than term patients of superficial SSI (0.9% vs. 0.7%), wound dehiscence (1.2% vs. 0.7%), deep space infections (2.1% vs. 0.9%), UTIs (1.2% vs. 0.5%), and renal insufficiency (0.5% vs. 0%), although these failed to reach significance (*p* > 0.05, all).

When multivariate regression analysis was performed, prematurity was a predictor for overall infection (OR: 2.4, 95% CI: 1.03–5.5, *p* = 0.043), but not a significant predictor of total complications, readmissions, reoperations, deep space infections, or superficial SSI or wound dehiscence (Table 5). When evaluating comorbidities, baseline cognitive impairment was a predictor for total complications (OR: 4.3, 95% CI: 1.7–10.6, *p* = 0.002) and reoperations (OR: 4.0, 95% CI: 1.3–12.8, *p* = 0.018), but not readmissions (OR: 3.0, 95% CI: 0.7–12.5, *p* = 0.142). Neuromuscular disease, cerebral palsy, structural pulmonary/airway abnormality, asthma, chronic lung disease, seizure disorder, esophageal/GI disease, and previous cardiac surgery were not significant predictors for 30-day complications, readmissions, or reoperations.

When subgroups of prematurity were analyzed and compared to the 5099 patients born at term, the very premature cohort had a greater 30-day complication rate than the term, moderate-to-late premature, and extremely premature cohorts (9.9% vs. 2.9%, 5.6%, and 3.7%, respectively; *p* < 0.001) (Table 6). Specifically, very premature patients experienced the highest rate of superficial incisional SSIs (2.0%), dehiscence (3.0%), and UTIs (3.0%; all *p* < 0.004). Moderate-to-late premature patients had the highest rate of deep space infection (2.4%) and renal insufficiency (0.8%; all *p* < 0.009). Furthermore, the very premature cohort experienced greater 30-day reoperation rates than the extremely premature, moderate-to-late premature, and term cohorts (5.9% vs. 2.5%, 4.8%, and 2.2%, respectively; *p* = 0.007). Readmission rates within the 30-day postoperative period increased with increasing degree of prematurity (term: 3.1%, moderate-to-late premature: 8.3%, very premature: 14.3%, extremely premature: 18.2%; *p* < 0.001).

When multivariate regression analysis was performed, very premature birth history predicted UTIs, superficial SSI/wound dehiscence, and any infection (OR = 9.8, 4.4, and 4.4), and moderate-to-late premature predicted renal insufficiency, deep space infections, and any infection (OR = 9.7, 5.2, and 3.3), all *p* < 0.05. No associations were identified between extreme prematurity at birth and 30-day adverse outcomes (Table 7).

## 4. Discussion

Adolescent idiopathic scoliosis is the most common form of scoliosis, with prevalence estimates of up to 5.2% of the population [9]. Although AIS has been shown to have an association with premature birth, postoperative complications following spinal fusions in this population are not well studied. In this investigation, prematurity was identified as a predictor for 30-day complications, readmissions, and reoperations in AIS patients undergoing PSF.

Surgical correction of AIS has reported complication rates of up to 15.4% [23]. Other studies have more specifically shown infection rates in patients undergoing spinal fusion for AIS ranging from 0.71% to 6.9% [23,24,25,26]. In this study, comparing propensity score-matched cohorts, patients born at term had a total complication rate of 4.2% within the 30-day postoperative period, while premature patients had a total complication rate of 6.3%, although this failed to reach significance (*p* = 0.168). The current study identified that prematurity was a significant predictor of overall infection in patients undergoing PSF for AIS, with an odds ratio of 2.4 (95% CI: 1.03–5.5, *p* = 0.043). Furthermore, when premature patients were sub-analyzed according to degrees of prematurity, very premature was a predictor for overall infection, superficial surgical site infection, and UTIs, while moderate-to-late prematurity was a predictor for overall infection, deep space infections, and renal insufficiency.

A possible explanation for the association between prematurity and infection risk is the burden of comorbidities in this population. Premature birth has a well-studied association with various comorbidities, including but not limited to cardiac, pulmonary, neurological, and cognitive deficiencies, as we expectedly observed the preterm cohorts to possess greater rates of comorbidities than the cohort born at term [6,7,8,19,27,28]. The comorbidity rates were especially pronounced in the extremely premature subgroup of prematurity, which possessed significantly higher rates of asthma, chronic lung disease, structural pulmonary abnormality, esophageal/GI disease, cognitive impairment, cerebral palsy, structural CNS abnormality, and neuromuscular disorders than the very premature, moderate-to-late premature, and term cohorts. Subsequently, the present study expectedly found the Premature cohort to have a significantly higher average ASA classification than term patients. Past studies have specifically related ASA classification to the risk of developing a surgical site infection after PSF in pediatric patients [29,30]. The higher average comorbidity burden and ASA scores in the premature cohort likely contributed to the higher rate of total infections and readmissions compared to the propensity score-matched cohort born at term. While no individual comorbidity was associated with an increased risk of complications, the overall increased comorbidity burden may contribute to these findings. The higher postoperative infection rate is potentially due to inherent deficiencies in the immune response of premature individuals, as premature birth is known to be associated with underdeveloped immune systems, limiting a newborn’s potential to combat pathogens and infections, with evidence suggesting this population continues to have limitations in immune response throughout life when compared to individuals born at term [31,32,33,34].

Of the baseline comorbidities examined in the current study, baseline cognitive impairment was a predictor for complications (OR = 4.3) and reoperations (OR = 4.0) (all *p* < 0.05). The study also found that neuromuscular disease, cerebral palsy, structural pulmonary/airway abnormality, asthma, bronchopulmonary dysplasia/chronic lung disease, seizure disorder, esophageal/GI disease, and previous cardiac surgery were not significant predictors for complications, readmissions, or reoperations in our patient population. Studies have suggested that there may be an increased prevalence of scoliosis in patients with neurological and/or cognitive impairment, potentially due to deficiencies in muscle tone [35,36]. Cerebral palsy, specifically, has been shown to have a high association with scoliosis with subsequently high rates of complications during corrective surgeries [37,38]. The current findings suggest that cerebral palsy in itself might not be a predictor for increased complication rates in premature patients with AIS who underwent PSF, but rather that the increased complication rates may be due to other comorbidities affecting this population. Furthermore, several studies have demonstrated increased incidences of scoliosis in pediatric patients who underwent thoracic surgeries, particularly reconstructive cardiothoracic or esophageal surgeries, although the management and outcomes of these patients’ scoliosis were rarely examined [11,39,40].

Since the existing literature shows that a higher number of levels being fused is a predictor of increased complications, it was important that this was accounted for in the propensity score-match of premature and term cohorts [30,41]. When premature subgroups were analyzed, increasing rates of ≥13-level PSFs were found with increasing degrees of prematurity, with only the extremely premature cohort experiencing higher rates of ≥13-level PSFs. The study did find the extremely premature cohort to experience the highest rate of readmission, but the very premature cohort experienced the highest rate of total complications and reoperations. Further studies are ultimately warranted to explore the relationship between the extent of level fusion and complications in this specific patient population.

This study has limitations consistent with any study of a retrospective nature. Furthermore, study data were limited to the NSQIP participating institutions, which does not represent the entire AIS premature population undergoing PSF, and the outcome variables were restricted to the 30-day postoperative follow-up, potentially missing complications arising beyond that interval of time. For example, Bartley et al. [42] reported a 2.6% rate of major complications within six weeks of PSF for AIS, and a 4.1% rate of major complications within two years and reported that the majority of the delayed complications were related to the wound or instrumentation. There are also limitations inherent to NSQIP studies, namely inconsistencies in the coding of diagnoses and procedures as well as in the exclusion of patient factors that may be clinically relevant to this study. For example, curve magnitude and curve type were not available, as no radiographic data is included in the NSQIP; thus, this could not be analyzed as a potential predictor of complication rates due to limitations with the database. All patients who were included in this study were diagnosed with AIS, with the associated coding, at the primary reporting centers by their respective treating surgeons; an assumption must be made that this is truly AIS, without the ability to confirm this with clinical or radiographic data; thus, this serves as a limitation of this study, as there is a degree of baseline comorbidities that overlap with the neuromuscular scoliosis patient population. Additionally, the limited sample size available for this study for the extreme prematurity cohort potentially underpowered its analysis. Despite these limitations, our study provides valuable information by highlighting prematurity at birth as a risk factor for short-term postoperative complications for AIS patients undergoing PSF.

This study found prematurity at birth was associated with higher rates of 30-day postoperative readmissions as well as a predictor for postoperative infections in AIS patients who underwent posterior spinal fusions. It also found that baseline cognitive impairment was a predictor of total complications and reoperations in this patient population. Recognizing prematurity and associated comorbidities as risk factors for postoperative complications may better allow spine surgeons and medical providers to optimize this patient population in the perioperative period to potentially result in more favorable outcomes.

## Figures and Tables

**Table 1:** Demographics of premature and term AIS patients who underwent PSF from 2012–2016 propensity-score matched for age, sex, and levels fused.

Demographics		Premature	Term	*p*-Value
Number		432	432	
Age (years)		14.1	14.4	0.071
Sex	Male	32.4%	35.6%	0.315
	Female	67.6%	64.4%	
Race	White	76.5%	80.1%	0.109
	Black	21.8%	16.9%	
	Other	1.7%	3.1%	

**Table 2:** Baseline comorbidities of propensity-score matched premature and term AIS patients who underwent PSF from 2012–2016. Bold values are statistically significant (*p* < 0.05).

Baseline Comorbidities	Premature	Term	*p*-Value
Neuromuscular Disorder	30.1%	22.7%	**0.014**
Asthma	12.5%	5.8%	**0.001**
Chronic Lung Disease	7.4%	1.9%	**<0.001**
Structural Pulmonary/Airway Abnormalities	6.7%	3.9%	0.069
Esophageal/Gastrointestinal Disease	15.0%	7.2%	**<0.001**
Previous Cardiac Surgery	7.6%	1.4%	**<0.001**
Seizure Disorder	14.8%	5.3%	**<0.001**
Structural CNS Abnormality	19.7%	7.6%	**<0.001**
Cerebral Palsy	22.7%	4.9%	**<0.001**
Cognitive Impairment	36.3%	11.6%	**<0.001**

**Table 3:** Baseline comorbidities, hospital parameters, and outcomes across term (≥37 weeks), and preterm (includes MLP, VP, and EP) patients. Bold values are statistically significant (*p* < 0.05).

Operative and Hospital Related Parameters	Term	Preterm	*p*-Value
**ASA Classification**			
I	20.6%	8.3%	**<0.001**
II	58.6%	53.7%	
III	19.0%	36.1%	
IV	1.9%	1.6%	
V	0%	0%	
**Operative Time (minutes)**	296.3	305.1	0.252
**Hospital Length of Stay (days)**	5.6	6.5	**0.045**
**Fusion Length**			
7–12 Levels	60.6%	56.7%	0.240
≥13 Levels	39.4%	43.3%	
**30-Day Complications**	4.2%	6.3%	0.168
Superficial Incisional SSI	0.7%	0.9%	0.704
Wound Dehiscence	0.7%	1.2%	0.477
Deep Space Infections	0.9%	2.1%	0.162
Pneumonia	0.9%	0.2%	0.178
Urinary Tract Infection	0.5%	1.2%	0.255
Renal Insufficiency	0%	0.5%	0.157
**30-Day Reoperations**	2.3%	4.6%	0.063
**30-Day Readmissions**	3.2%	11.0%	**0.003**

**Table 4:** Baseline comorbidities, hospital parameters, and outcomes across term (≥37 weeks), and moderate-to-late prematurity (MLP, 32 ≤ x < 37 weeks), very premature (VP, 28 ≤ x < 32 weeks), and extremely premature (EP, <28 weeks). Bold values are statistically significant (*p* < 0.05).

	Term	Moderate-to-Late Premature	Very Premature	Extremely Premature	*p*-Value
**Number of Patients**	5099	250	101	81	
**Baseline Comorbidities**					
Neuromuscular Disorder	14.5%	23.2%	35.6%	44.4%	**<0.001**
Asthma	7.3%	10.0%	12.9%	19.8%	**<0.001**
Chronic Lung Disease	1.6%	4.8%	4.0%	19.8%	**<0.001**
Structural Pulmonary/Airway Abnormalities	3.0%	6.8%	4.0%	9.9%	**<0.001**
Esophageal/GI Disease	5.4%	12.4%	14.9%	23.5%	**<0.001**
Previous Cardiac Surgery	2.2%	7.2%	8.9%	7.4%	**<0.001**
Seizure Disorder	5.0%	9.6%	22.8%	21.0%	**<0.001**
Structural CNS Abnormality	8.9%	13.6%	24.8%	32.1%	**<0.001**
Cerebral Palsy	4.0%	11.6%	31.7%	45.7%	**<0.001**
Cognitive Impairment	11.0%	27.2%	41.6%	58.0%	**<0.001**
**ASA Classification**					
1	22.9%	8.4%	6.9%	9.9%	**<0.001**
2	58.9%	61.2%	48.5%	37.0%	
3	17.3%	28.8%	41.6%	51.9%	
4	0.8%	1.2%	3.0%	1.2%	
5	0.0%	0.0%	0.0%	0.0%	
**Operative Time (minutes)**	278.2	295.2	310.2	329.3	**<0.001**
**Fusion Length**					
7–12 Levels	69.8%	60.0%	56.4%	46.9%	**<0.001**
≥13 Levels	30.2%	40.0%	43.6%	53.1%	
**Length of Stay (days)**	5.1	4.9	5.9	6.5	**0.040**

**Table 5:** Prematurity and baseline cognitive impairment, neuromuscular disease, cerebral palsy, previous cardiac surgery, structural pulmonary/airway abnormality, asthma, chronic lung disease, seizure disorder, and esophageal/GI disease) as predictive factors for 30-day postoperative complications, readmission, and reoperations in propensity-score matched premature and term patients who underwent PSF for AIS. Bold values are statistically significant (*p* < 0.05).

Multivariate Regression Analysis	OR	95% CI	*p*-Value
**Premature**			
Total Complications	0.8	0.4–1.6	0.460
Readmissions	2.0	0.7–6.1	0.218
Reoperation	1.1	0.5–2.7	0.834
Overall Infection	2.4	1.03–5.5	**0.043**
Deep Space Infections	3.1	0.98–9.7	0.055
Superficial SSI or Dehiscence	1.3	0.4–3.8	0.633
**Cognitive Impairment**			
Total Complications	4.3	1.7–10.6	**0.002**
Readmissions	3.0	0.7–12.5	0.142
Reoperation	4.0	1.3–12.8	**0.018**

**Table 6:** Postoperative outcomes across Term (≥37 weeks), and moderate-to-late prematurity (32 ≤ x < 37 weeks), very premature (28 ≤ x < 32 weeks), and extremely premature (<28 weeks). Difference in superscript letters indicates inter-group differences (*p* < 0.05). Bold values are statistically significant (*p* < 0.05).

	Term	Moderate-to-Late Premature	Very Premature	Extremely Premature	*p*-Value
**Number of Patients**	5099	250	101	81	
**30-Day Complications**	2.9% ^a^	5.6% ^a,b^	9.9% ^b^	3.7% ^a,b^	**<0.001**
Superficial Incisional SSI	0.5% ^a^	0.8% ^a^	2.0% ^a^	0.0% ^a^	0.194
Wound Dehiscence	0.5% ^a^	0.4% ^a,b^	3.0% ^b^	1.2% ^a,b^	**0.004**
Deep Space Infection	0.6% ^a^	2.4% ^b^	2.0% ^a,b^	1.2% ^a,b^	**0.002**
Pneumonia	0.6% ^a^	0.0% ^a^	1.0% ^a^	0.0% ^a^	0.523
Urinary Tract Infection	0.4% ^a^	0.8% ^a,b^	3.0% ^b^	0.0% ^a,b^	**0.001**
Renal Insufficiency	0.1% ^a^	0.8% ^b^	0.0% ^a,b^	0.0% ^a,b^	**0.009**
**30-Day Reoperations**	2.2% ^a^	4.8% ^b^	5.9% ^a,b^	2.5% ^a,b^	**0.007**
**30-Day Readmissions**	3.1% ^b^	8.3% ^a,b^	14.3% ^a,b^	18.2% ^b^	**0.002**

**Table 7:** Extremely premature (<28 weeks), very premature (28 ≤ x < 32 weeks), and moderate-to-late prematurity (32 ≤ x < 37 weeks) as predictive factors for 30-day postoperative complications, readmission, and reoperations in premature patients who underwent PSF for AIS. Bold values are statistically significant (*p* < 0.05).

Multivariate Regression Analysis	OR	95% CI	*p*-Value
**Extremely Premature**			
Total Complications	0.4	0.1–1.4	0.144
Readmissions	4.3	0.7–27.6	0.126
Reoperation	0.5	0.1–2.3	0.399
Overall Infection	1.9	0.5–8.0	0.377
Deep Space Infections	4.0	0.9–17.2	0.062
Superficial Infection or Dehiscence	0.7	0.2–19.9	0.757
**Very Premature**			
Total Complications	1.6	0.7–3.3	0.250
Readmissions	3.1	0.5–20.8	0.249
Reoperation	1.5	0.6–3.8	0.351
Overall Infection	4.4	1.9–10.5	**<0.001**
Deep Space Infections	3.0	0.7–12.7	0.143
Superficial Infection or Dehiscence	4.4	1.5–12.7	**0.006**
Urinary Tract Infection	9.8	2.8–34.3	**<0.001**
**Moderate-to-Late Prematurity**			
Total Complications	1.2	0.6–2.2	0.613
Readmissions	1.7	0.5–5.9	0.381
Reoperation	1.6	0.8–3.0	0.173
Overall Infection	3.3	1.7–6.3	**<0.001**
Deep Space Infections	5.2	2.2–11.4	**<0.001**
Superficial Infection or Dehiscence	1.3	0.4–4.2	0.663
Renal Insufficiency	9.7	1.8–53.7	**0.009**
Urinary Tract Infection	2.4	0.5–10.3	0.253

## Data Availability

Publicly available datasets were analyzed in this study, which can be obtained via the ACS.

## References

[1] Frey H.A., Klebanoff M.A. (2016). The epidemiology, etiology, and costs of preterm birth. Semin. Fetal Neonatal Med..

[2] Mangham L.J., Petrou S., Doyle L.W., Draper E.S., Marlow N. (2009). The Cost of Preterm Birth Throughout Childhood in England and Wales. Pediatrics.

[3] Johnston K.M., Gooch K., Korol E., Vo P., Eyawo O., Bradt P., Levy A. (2014). The economic burden of prematurity in Canada. BMC Pediatr..

[4] Kwinta P., Pietrzyk J.J. (2010). Preterm birth and respiratory disease in later life. Expert Rev. Respir. Med..

[5] Bavineni M., Wassenaar T.M., Agnihotri K., Ussery D.W., Lüscher T.F., Mehta J.L. (2019). Mechanisms linking preterm birth to onset of cardiovascular disease later in adulthood. Eur. Heart J..

[6] Axelrod D.M., Chock V.Y., Reddy V.M. (2016). Management of the Preterm Infant with Congenital Heart Disease. Clin. Perinatol..

[7] Kumar K.R., Clark D.A., Kim E.M., Perry J.D., Wright K., Thomas S.A., Thompson E.J., Greenberg R.G., Smith P.B., Benjamin D.K. (2018). Association of Atrial Septal Defects and Bronchopulmonary Dysplasia in Premature Infants. J. Pediatr..

[8] Chu P.Y., Li J.S., Kosinski A.S., Hornik C.P., Hill K.D. (2017). Congenital Heart Disease in Premature Infants 25-32 Weeks’ Gestational Age. J. Pediatr..

[9] Konieczny M.R., Senyurt H., Krauspe R. (2013). Epidemiology of adolescent idiopathic scoliosis. J. Child. Orthop..

[10] Fadzan M., Bettany-Saltikov J. (2017). Etiological Theories of Adolescent Idiopathic Scoliosis: Past and Present. Open Orthop. J..

[11] Mery C.M., Guzmán-Pruneda F.A., De León L.E., Zhang W., Kuo F.H., Carpenter K.L., Stringer L.D., Adachi I., Heinle J.S., McKenzie E.D. (2018). Risk Factors for Development and Progression of Scoliosis after Pediatric Cardiothoracic Operations. Ann. Thorac. Surg..

[12] Grivas T., Dangas S., Polyzois B.D., Samelis P. (2002). The Double Rib Contour Sign (DRCS) in lateral spinal radiographs: Aetiologic implications for scoliosis. Stud. Health Technol. Inform..

[13] Burwell R.G., Aujla R.K., Grevitt M.P., Dangerfield P.H., Moulton A., Randell T.L., Anderson S.I. (2009). Pathogenesis of adolescent idiopathic scoliosis in girls—A double neuro-osseous theory involving disharmony between two nervous systems, somatic and autonomic expressed in the spine and trunk: Possible dependency on sympathetic nervous system and hormones with implications for medical therapy. Scoliosis.

[14] Nachemson A.L., Sahlstrand T. (1977). Etiologic Factors in Adolescent Idiopathic Scoliosis. Spine.

[15] Asher M.A., Burton D.C. (2006). Adolescent idiopathic scoliosis: Natural history and long term treatment effects. Scoliosis.

[16] Kotwicki T., Chowanska J., Kinel E., Czaprowski D., Janusz P., Tomaszewski M. (2013). Optimal management of idiopathic scoliosis in adolescence. Adolesc. Health Med. Ther..

[17] Helenius I. (2013). Anterior surgery for adolescent idiopathic scoliosis. J. Child. Orthop..

[18] Geck M.J., Rinella A., Hawthorne D., Macagno A., Koester L., Sides B., Bridwell K., Lenke L., Shufflebarger H. (2009). Comparison of Surgical Treatment in Lenke 5C Adolescent Idiopathic Scoliosis: Anterior Dual Rod Versus Posterior Pedicle Fixation Surgery: A comparison of two practices. Spine.

[19] Desai J., Aggarwal S., Lipshultz S., Agarwal P., Yigazu P., Patel R., Seals S., Natarajan G. (2017). Surgical Interventions in Infants Born Preterm with Congenital Heart Defects: An Analysis of the Kids’ Inpatient Database. J. Pediatr..

[20] Howard P.J., Worrell C.H. (1952). Premature infants in later life; study of intelligence and personality of 22 premature infants at ages 8 to 19 years. Pediatrics.

[21] Wynne-Davies R. (1975). Infantile idiopathic scoliosis. Causative factors, particularly in the first six months of life. J. Bone Jt. Surg. Br..

[22] Talasila S.S.A., Gorantla M., Thomas V. (2017). A study on screening for scoliosis among school children in the age group of 10-14 using a cost effective and an innovative technique. Int. J. Community Med. Public Health.

[23] Carreon L.Y., Puno R.M., Lenke L.G., Richards B.S., Sucato D.J., Emans J.B., Erickson M.A. (2007). Non-Neurologic Complications Following Surgery for Adolescent Idiopathic Scoliosis. J. Bone Jt. Surg. Am..

[24] Kamath V.H.D., Cheung J.P.Y., Mak K.C., Wong Y.W., Cheung W.Y., Luk K.D.K., Cheung K.M.C. (2016). Antimicrobial prophylaxis to prevent surgical site infection in adolescent idiopathic scoliosis patients undergoing posterior spinal fusion: 2 doses versus antibiotics till drain removal. Eur. Spine J..

[25] Coe J.D., Arlet V., Donaldson W., Berven S., Hanson D.S., Mudiyam R., Perra J.H., Shaffrey C.I. (2006). Complications in Spinal Fusion for Adolescent Idiopathic Scoliosis in the New Millennium. A Report of the Scoliosis Research Society Morbidity and Mortality Committee. Spine.

[26] Rihn J.A., Lee J.Y., Ward W.T. (2008). Infection after the Surgical Treatment of Adolescent Idiopathic Scoliosis: Evaluation of the diagnosis, treatment, and impact on clinical outcomes. Spine.

[27] Davidson L.M., Berkelhamer S.K. (2017). Bronchopulmonary Dysplasia: Chronic Lung Disease of Infancy and Long-Term Pulmonary Outcomes. J. Clin. Med..

[28] Manchego P.A., Cheung M., Zannino D., Nunn R., D’Udekem Y., Brizard C. (2018). Audit of Cardiac Surgery Outcomes for Low Birth Weight and Premature Infants. Semin. Thorac. Cardiovasc. Surg..

[29] Linam W.M., Margolis P.A., Staat M.A., Britto M.T., Hornung R., Cassedy A., Connelly B.L. (2009). Risk Factors Associated with Surgical Site Infection after Pediatric Posterior Spinal Fusion Procedure. Infect. Control Hosp. Epidemiol..

[30] Rao S.B., Vasquez G., Harrop J., Maltenfort M., Stein N., Kaliyadan G., Klibert F., Epstein R., Sharan A., Vaccaro A. (2011). Risk Factors for Surgical Site Infections Following Spinal Fusion Procedures: A Case-Control Study. Clin. Infect. Dis..

[31] Melville J.M., Moss T.J.M. (2013). The immune consequences of preterm birth. Front. Neurosci..

[32] Catania V.D., Boscarelli A., Lauriti G., Morini F., Zani A. (2019). Risk Factors for Surgical Site Infection in Neonates: A Systematic Review of the Literature and Meta-Analysis. Front. Pediatr..

[33] Perin M.C.A.A., Schlindwein C.F., de Moraes-Pinto M.I., Simão-Gurge R.M., Mimica A.F.D.M.A., Goulart A.L., dos Santos A.M.N. (2012). Immune response to tetanus booster in infants aged 15 months born prematurely with very low birth weight. Vaccine.

[34] Kirmani K.I., Lofthus G., Pichichero M.E., Voloshen T., D’Angio C.T. (2002). Seven-Year Follow-up of Vaccine Response in Extremely Premature Infants. Pediatrics.

[35] Downs J., Torode I., Wong K., Ellaway C., Elliott E., Christodoulou J., Jacoby P., Thomson M.R., Izatt M., Askin G. (2016). The Natural History of Scoliosis in Females with Rett Syndrome. Spine.

[36] Gu Y., Shelton J.E., Ketchum J.M., Cifu D.X., Palmer D., Sparkman A., Jermer-Gu M.K., Mendigorin M. (2011). Natural History of Scoliosis in Nonambulatory Spastic Tetraplegic Cerebral Palsy. PM R.

[37] McCarthy J.J., D’andrea L.P., Betz R.R., Clements D.H. (2006). Scoliosis in the Child with Cerebral Palsy. J. Am. Acad. Orthop. Surg..

[38] Broom M.J., Banta J.V., Renshaw T.S. (1989). Spinal fusion augmented by luque-rod segmental instrumentation for neuromuscular scoliosis. J. Bone Jt. Surg..

[39] Sistonen S.J., Helenius I., Peltonen J., Sarna S., Rintala R.J., Pakarinen M.P. (2009). Natural History of Spinal Anomalies and Scoliosis Associated with Esophageal Atresia. Pediatrics.

[40] Van Biezen F.C., Bakx P.A., De Villeneuve V.H., Hop W.C. (1993). Scoliosis in children after thoracotomy for aortic coarctation. J. Bone Jt. Surg..

[41] Deyo R.A., Mirza S.K., Martin B.I., Kreuter W., Goodman D.C., Jarvik J.G. (2010). Trends, Major Medical Complications, and Charges Associated with Surgery for Lumbar Spinal Stenosis in Older Adults. JAMA.

[42] Bartley C.E., Yaszay B., Bastrom T.P., Shah S.A., Lonner B.S., Asghar J., Miyanji F., Samdani A., Newton P.O. (2017). Perioperative and Delayed Major Complications Following Surgical Treatment of Adolescent Idiopathic Scoliosis. J. Bone Jt. Surg. Am..

